# Neuronal Cell Death and Degeneration through Increased Nitroxidative Stress and Tau Phosphorylation in HIV-1 Transgenic Rats

**DOI:** 10.1371/journal.pone.0169945

**Published:** 2017-01-20

**Authors:** Young-Eun Cho, Myoung-Hwa Lee, Byoung-Joon Song

**Affiliations:** 1 Section of Molecular Pharmacology and Toxicology, Laboratory of Membrane Biochemistry and Biophysics, National Institute on Alcohol Abuse and Alcoholism, Bethesda, Maryland, United States of America; 2 Office of the Clinical Director, National Institute of Neurological Disorders and Stroke, Bethesda, Maryland, United States of America; Rutgers University, UNITED STATES

## Abstract

The underlying mechanisms for increased neurodegeneration and neurocognitive deficits in HIV-infected people are unclear. Therefore, this study was aimed to investigate the mechanisms of increased neurodegeneration in 5-month old male HIV-1 Transgenic (Tg) rats compared to the age- and gender-matched wild-type (WT) by evaluating histological changes and biochemical parameters of the key proteins involved in the cell death signaling and apoptosis. Histological and immunohistochemical analyses revealed decreased neuronal cells with elevated astrogliosis in HIV-1 Tg rats compared to WT. Mechanistic studies revealed that increased levels of nitroxidative stress marker proteins such as NADPH-oxidase, cytochrome P450-2E1 (CYP2E1), inducible nitric oxide synthase (iNOS), the stress-activated mitogen-activated protein kinases such as JNK and p38K, activated cell-cycle dependent CDK5, hypoxia-inducible protein-1α, nitrated proteins, hyperphosphorylated tau, and amyloid plaques in HIV-Tg rats were consistently observed in HIV-1 Tg rats. Confocal microscopy and cell viability analyses showed that treatment with an antioxidant *N*-acetylcysteine or a specific inhibitor of iNOS 1400W significantly prevented the increased apoptosis of neuro-2A cells by HIV-1 Tat or gp120 protein, demonstrating the causal role of HIV-1 mediated nitroxidative stress and protein nitration in promoting neuronal cell death. Immunoprecipitation and immunoblot analysis confirmed nitration of Hsp90, evaluated as an example of nitrated proteins, suggesting possible involvement of nitrated proteins in neuronal damage. Further, activated p-JNK directly binds tau and phosphorylates multiple amino acids, suggesting an important role of p-JNK in tau hyperphosphorylation and tauopathy. These changes were accompanied with elevated levels of many apoptosis-related proteins Bax and cleaved (activated) caspase-3 as well as proinflammatory cytokines including TNF-α, IL-6 and MCP-1. Collectively, these results indicate that raised nitroxidative stress accompanied by elevated inflammation, cell death signaling pathway including activated p-JNK, C-terminal C99 amyloid fragment formation and tau hyperphosphorylation are responsible for increased apoptosis of neuronal cells and neurodegeneration in 5-month old HIV-Tg rats.

## Introduction

Infection with human immunodeficiency virus type1 (HIV-1) is associated with a variety of pathological conditions such as central and peripheral neuropathies [1], lymphoid depletion [2], interstitial pneumonia [3], tubulointerstitial nephritis [4], intestinal disturbance [5], heart disease [6], and liver diseases often resulting in death [7]. HIV-1 infection negatively affects the central nervous system (CNS) accompanied with neurological complications such as behavioral and cognitive deficits, contributing to dementia, as known as HIV-associated neurocognitive disorders (HAND) and HIV-associated dementia (HAD), as observed in a subset of HIV-infected people [8, 9].

The neuropathological consequences of HIV-1 infection are well-characterized. For instance, cognitive decline, neuronal damage, and loss of nerve cells from the neuroinflammatory process and elevated oxidative stress could be observed following HIV-1 infection in experimental animal models or cultured cells [10–12]. In fact, HIV infections into the CNS have also been reported to promote neuronal calcium dysregulation, mitochondrial damage, oxidative stress [13], and caspase-3 dependent apoptosis [14]. In addition, the increased inflammatory response to HIV-1 viral proteins or cytokines can further increase oxidative stress and facilitate the apoptosis signaling cascade [10, 15].

Recent studies have shown that hyper-phosphorylation of tau aggregates and neurofibrillary tangles (NFTs) may play an important role in the Alzheimer’s disease and Parkinson’s disease [16, 17]. Inflammatory stimuli can promote tau phosphorylation, leading to hyperphosphorylation of tau, which has been associated with neurodegeneration [18, 19]. Previous reports also showed that HIV-associated neurocognitive disorders had higher levels of hyperphosphorylated tau protein in HIV-1 infected individuals [20]. However, the source of increased nitroxidative stress and the role of mitogen-activated protein kinases (MAPKs), especially cell-death associated JNK and p38K, in tau phosphorylation and neuronal cell death have not been clearly elucidated in HIV-1 infected people or animal models.

Therefore, this study was aimed to investigate the molecular mechanisms of increased neurodegeneration in HIV-1 Tg rats, as a surrogate model for HIV-infected people, compared to the age- and gender-matched WT counterparts. For this purpose, we specifically examined the source and levels of increased nitroxidative stress, changes in the apoptosis signaling pathways and the role of MAPKs including activated p-JNK in stimulating tau phosphorylation and neuronal cell death in the hippocampus of 5-month old HIV-1 Tg rats and the corresponding WT.

## Material and Methods

### Materials

All chemicals, including anti-CT19 amyloid antibody and 10% neutral buffered formalin solution used in this study, were from Sigma Chemical (St. Louis, MO, USA). Specific antibodies to MAPKs, phospho-MAPKs, total tau, p-tau-T181, p-tau-S396, β-amyloid, TNF-α, MCP-1, GFAP, cleaved caspase-3, or IL-6 were purchased from Cell Signaling (Danver, MA, USA). Specific antibody to GSK-3β, p-GSK-3β-S9, CDK5, p-tau-T231, iNOS, 3-NT, CYP2E1, or NeuN was from Abcam (Cambridge, MA, USA). Antibody against p-CDK5-Tyr15, cleaved caspase-3, Bax, GAPDH, NeuN, GFAP, HSP90, HNF-1α, BNIP3, and secondary antibodies conjugated with horse radish peroxidase were obtained from Santa Cruz Biotechnology (Santa Cruz, CA, USA). Recombinant HIV-1 Tat and gp120 (B.MN D11 strain) proteins were provided by the NIAID AIDS Reagent Program (National Institutes of Health, Bethesda, MD, USA). Neuro-2a cells were purchased from the ATCC (American Type Culture Cells (Manassas, VA). Other materials not described here were the highest grades available and/or the same, as recently described [21, 22].

### Animals

Five-month old male HIV-1 Tg and F344/NHsd wild-type (WT) control rats (n = 4~8/group) were purchased from Harlan-Enviro Laboratories (Indianapolis, IN). All rats were maintained under controlled lighting (12-h light/dark cycle) with food and water provided *ad libitum*. The HIV-1 Tg rats show normal appearance except for cataract and smaller body weights (Figure A in S1 File) compared to the age- and gender-matched WT rats. Individual WT and HIV-1 Tg rats were briefly sedated by exposure to carbon dioxide gas and immediately decapitated to rapidly collect brains and trunk blood into heparinized tubes. The half of the brain of each rat was fixed in 10% neutral buffered formalin solution while the other hemi-brain was quickly frozen with dry ice powder for biochemical analyses. In some rats, we also collected the whole brains, which were rapidly fixed in neutral buffered formalin solution for coronal sections to perform Congo red staining and IHC analyses. All studies were completed in compliance with the protocols approved by the NIH/NIAAA Animal Care and Use Committee.

### Cell culture and viability assay

Mouse neuroblastoma neuro-2a (N2a; CCL-131, American Type Culture Collection, Manassas, VA) cells were grown in Dulbecco's Modified Eagle Medium (DMEM) supplemented with 10% heat inactivated fetal calf serum (FCS) and 1% penicillin-streptomycin. N2a cells in plastic culture dishes were maintained in a humidified atmosphere under 5% CO_2_ at 37°C. Cell viability was assessed by the metabolic reduction of 3-[4,5-dimethylthiazol-2-yl]-2,5-diphenyltetrazolium bromide (MTT). Briefly, cell proliferation assay was performed with N2a cells grown in 96-well microplates for 24 h. At 80% confluence, the N2a cells were exposed to the recombinant HIV-1 Tat (250 nM) or gp120 (100 nM) with or without an antioxidant *N*-acetylcysteine (NAC) (1 mM, Sigma) or a specific iNOS inhibitor 1400W (20 μM, Sigma) and incubated for additional 24 h. After replacing the media with 1x phosphate buffered saline (PBS), MTT solution (10 μL of 5 mg/mL in PBS into 0.1 mL final volume) was added to each well, and then incubated at 37°C for 3 h to allow the occurrence of formazan crystals, which were subsequently dissolved in DMSO. The absorbency of each sample well was read with an ELISA reader (Bio-Rad) at 570 nm. Cell viability rates were expressed relative to control values for each experiment.

### Enzyme-linked immunosorbent assay (ELISA)

Brain hippocampus lysates prepared from individual HIV-1 Tg rats or WT were analyzed by using the respective ELISA kits for TNF-α (Abcam) and MCP-1 (Abcam) by following the manufacturers' protocols. Lysates were prepared from the frozen hippocampus from each rat. The protein was extracted from the tissue using 1x RIPA buffer containing the protease inhibitor cocktail (Sigma). The lysates were then centrifuged at 13,000 rpm at 4°C for 30 min. The supernatants were then re-centrifuged for 20 min and the resultant supernatants collected. The protein concentration was measured with the BCA reagent (BioRad, Hercules, CA, USA) to use equal amounts of protein for the ELISA. Duplicate samples from each lysate (n = 4/group) were used for ELISA, which was repeated twice.

### Immunoprecipitation and immunoblot analyses

Immunoprecipitation of indicated proteins was carried out as described previously [21]. Briefly, one mg protein of hippocampus lysates was incubated with protein A/G-Sepharose-bound primary antibodies for 4 h at 4°C with constant head-to-tail rotations. Sepharose beads were pre-blocked with 2% BSA in PBS before being used for immunoprecipitation. After incubation, beads were washed twice with lysis buffer, four times with ice-cold PBS and mixed with 2x SDS-PAGE gel loading buffer. Immunoprecipitated protein samples were resolved by SDS-PAGE for subsequent immunoblot analyses using the antibody to each target protein, as indicated.

Equal amounts of hippocampus lysates were used for immunoblot analyses for the indicated proteins. The soluble protein lysates (0.05 or 0.1 mg protein/lane) were resolved on 10% SDS-PAGE gels and electrically transferred to nitrocellulose membranes. The membranes were then blocked with 3% skim milk in Tris-buffered saline with 0.5% Tween-20 (TBST) for 1 h at room temperature, and incubated with the primary antibody to each target protein overnight at 4°C. After removal of the primary antibody, the nitrocellulose membranes were washed with 1x PBS three times at 10-min intervals and incubated with the secondary anti-rat or -mouse IgG conjugated with horse radish peroxidase (Santa Cruz Biotechnology) for 1 h at room temperature. Image was developed using enhanced chemiluminescence detection reagent and the signal was detected on X-ray film. GAPDH was used as a loading control for hippocampus lysates. Density of each image was then digitally scanned and the band intensities on the scanned images were determined by using ImageJ software (National Institutes of Health, Bethesda, MD, USA).

### Immunohistochemistry and microscopy

Immunohistochemistry (IHC) staining for NeuN, GFAP, or cleaved caspase-3 was conducted with sections from formalin-fixed and paraffin-embedded brain specimens by standard procedures. Briefly, paraffin-embedded tissues were cut into 6-μm sections and transferred onto glass slides. Sections were deparaffinized with xylene three times for 5 min each and rehydrated in successive steps of 100% alcohol two times for 10 min, 95% alcohol two times for 10 min, and two rinses with distilled water for 5 min each. Pretreatment of antigens from paraffin sections was performed in hot 0.1 M sodium citrate buffer (pH 7.2) by using a microwave oven. These pretreated slides were cooled for 30 min at room temperature and rinsed with distilled water three times for 5 min each. After being rinsed in 1x PBS, the sections were pre-incubated with 2% skim milk for 1 h at room temperature and then incubated at 4°C overnight with the primary antibody to NeuN (1:300x), GFAP (1:300x), or cleaved caspase-3 (1:300x). After overnight incubation, the primary antibodies on slides were washed with 1x PBS three times for 5 min each and incubated in the dark for 2 h at room temperature with a secondary antibody conjugated with alkaline phosphatase (Dextran polymer from DAKO, Carpinteria, CA, USA). The slides were mounted in Vectashield mounting medium (H-1400; Vector Laboratories). Immunostained slides were analyzed with a microscope (Olympus, Tokyo, Japan), as shown (in Figure B in S1 File). Positively labeled cells were expressed as the number of cells per area selected in the region of interest (Figure C in S1 File).

### Measurements of reactive oxygen species (ROS) levels in plasma and NADPH oxidase activity

Total ROS levels in each plasma sample were measured using OxiSelect In Vitro ROS/RNS Assay Kit (Cell Biolabs, Inc.) following the manufacturer’s protocol. In brief, serum samples were diluted in 1x PBS (1:100), equilibrated at room temperature and then incubated for 15 min with stabilized dichlorodihydrofluorescein diacetate (DCFH-DA). Fluorescence of the oxidized compound was measured with a plate reader (BioTek, Winooski, VT, USA). The NADPH oxidase activity in 1 mg protein of individual hippocampal extracts was measured by using the NADP/NADPH Assay Kit (Abcam) according to the manufacturer's instructions to monitor the absorbance at 450 nm for the NADP/NADPH ratio on a microplate reader.

### Statistical analysis

All data shown in this study represent results from at least two independent experiments unless otherwise stated. Data are expressed as mean ± SD. Statistical analyses were performed using the Student's t-test and a *p* < 0.05 was considered significant. Other methods not described here were the same, as previously reported [21, 22].

## Results

### Brain neurodegeneration in HIV-1 Tg rats

**Fig 1:**
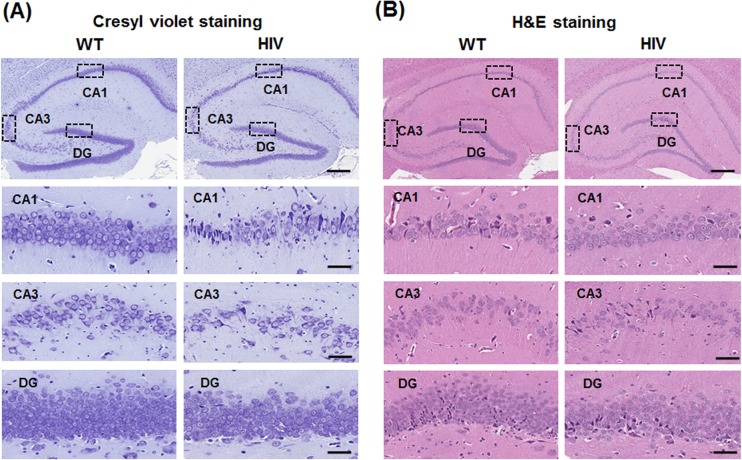
Hippocampal degeneration in HIV-1 Tg rats. (A, B) Representative images of the coronal hippocampal CA1 region, CA3 region and dentate gyrus (DG) from WT or HIV-1 Tg rats stained with cresyl violet (A) or H&E (B) to show decreased neuronal cells in HIV-1 Tg rats. Scale bar, 200 μm. (C, D) Densitometric analyses for both stainings (n = 4/group) are presented below. Data are expressed as mean ± SD, n = 4/group. *P < 0.05.

To verify HIV-1 induced neurodegeneration, we first compared the staining patterns of the brain tissues from 5-month old male HIV-1 Tg rats and the corresponding WT with using cresyl violet and hematoxylin and eosin (H&E). Cresyl violet (Fig 1A) and H&E staining (Fig 1B) results showed significantly increased neurodegeneration in the CA1 region, CA3 region and dentate gyrus (DG) of the hippocampus in HIV-1 Tg rats compared to those of the corresponding WT.

**Fig 2:**
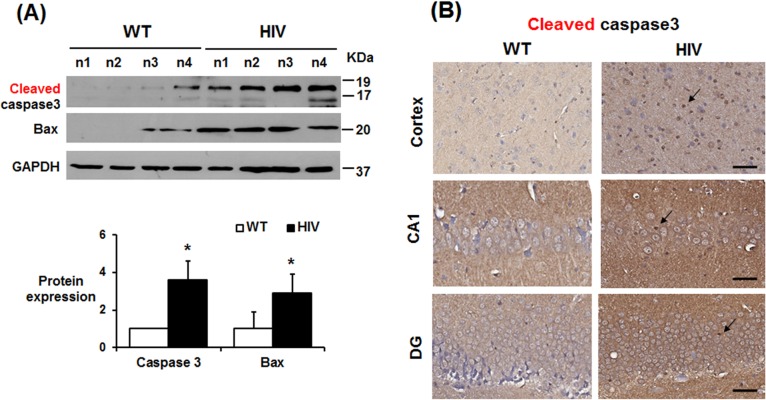
Increased apoptosis of neuronal cells in HIV-1 Tg rats. (A) Representative immunoblots of hippocampal lysates from WT and HIV-1 Tg rats with the respective antibody to cleaved (activated) caspase-3, Bax, or GAPDH, used as a loading control, as indicated. Densitometric analysis of the immunoblots for caspase-3 or Bax relative to GAPDH is shown below. Data are expressed as mean ± SD, *n* = 4/group. *P < 0.05. (B) Representative IHC images of cleaved caspase-3 in the cortex and hippocampal CA1 region from WT and HIV-1 Tg rats are shown. Arrows represent positively-stained cells with anti-cleaved caspase-3 antibody.

Immunoblot analyses also showed that the levels of cleaved (activated) caspase-3 and pro-apoptotic Bax were significantly elevated (i.e., by more than 3-fold) in the hippocampus of HIV-1 Tg rats compared to those of the WT (Fig 2A), suggesting mitochondria dependent apoptosis, as reported [23]. Consistently, the IHC data also showed that the number of cleaved caspase-3-positive cells, an apoptotic marker, was significantly increased in the hippocampus of the HIV-1 Tg rats compared to WT (Fig 2B).

### Increased gliosis and neuronal loss in HIV-1 Tg rats

**Fig 3:**
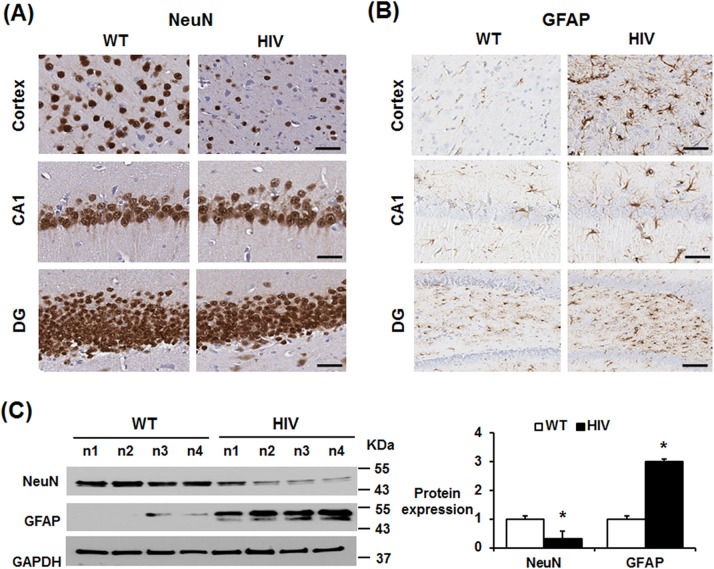
Decreased neuronal cells with increased astrocytes in HIV-1 Tg rats. (A, B) Representative images of the cortex, coronal hippocampal (CA1) region and dentate gyrus from WT or HIV-1 Tg rats stained with NeuN (A) or GFAP (B) to show decreased neuronal cells with increased astrocytes in HIV-1 Tg rats. Scale bar, 100 μm. (C) Immunoblots of hippocampal lysates from WT and HIV-1 Tg rats with the respective antibody to NeuN, GFAP, and GAPDH used as a loading control, are shown. Densitometric quantitation of the immunoblots for NeuN or GFAP relative to GAPDH (n = 4/group) is shown. *P < 0.05.

Neuronal cell loss and neurodegeneration are likely to increase the number of brain astrocytes while gliosis is a pathological marker of many neurodegenerative diseases. The IHC analysis, stained with the anti-NeuN antibody as a tool to identify neuronal cells [24], showed markedly decreased density of neuronal cells in the CA1 region and dentate gyrus of hippocampus and cortex region in HIV-1 Tg rats, compared to those of WT (Fig 3A, and Figure C in S1 File). In contrast, expression of GFAP, a marker of astrocytes activation, was markedly increased in the hippocampus of HIV-1 Tg rats than the WT counterparts (Fig 3B, Figures B and C in S1 File). The IHC results of neuronal loss with elevated gliosis in the hippocampus of HIV-1 Tg rats were further supported by immunoblot analysis with anti-NeuN (Fig 3C, top panel and Figure D in S1 File) or anti-GFAP antibody (Fig 3C, middle panel and Figure D in S1 File). Thus, these results strongly indicate impairment of neuronal function in 5-month old HIV-1 Tg male rats compared to the corresponding WT counterparts. Similarly, histological staining and IHC analyses showed decreased neuronal cell population with elevated GFAP expression in the hippocampus of 5-month old female HIV-1 Tg rats compared to those of gender- and age-matched WT (n≥5/group, data not shown).

### Elevated amyloid plaques and C-terminal C99 fragment in HIV-1 Tg rats

**Fig 4:**
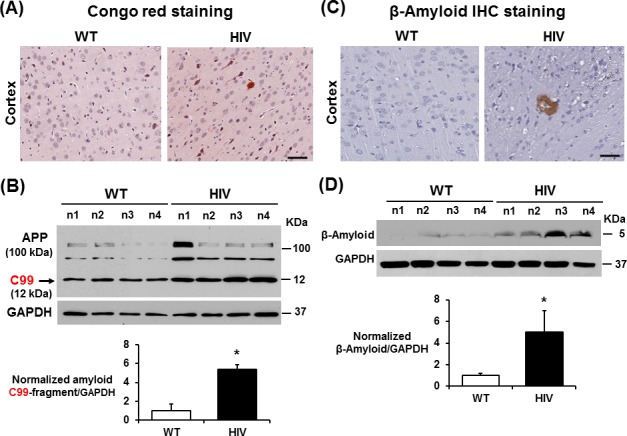
Increased production of amyloid plaques and C-terminal C99 fragment in the hippocampus of HIV-1 Tg rats. (A) Representative images of the cerebral cortex region stained with Congo red (A) to show different levels of amyloid plaques in WT and HIV-1 Tg rats. Scale bar, 200 μm. (B) Representative immunoblots for detecting APP and toxic amyloid C-terminal C99 fragment [25, 26] with the anti-CT19 antibody are shown. The densitometric results for the immunoblots (n = 4/group) are presented below. (C) Representative IHC images for β-amyloid in the cortex region from WT and HIV-1 Tg rat brains are shown. (D) Immunoblots of hippocampal lysates from WT and HIV-1 Tg rats with the respective antibody to β-amyloid or GAPDH used as a loading control, are shown. The densitometric results for the immunoblots (n = 4/group) are shown below. *P < 0.05.

We next examined the formation of amyloid plaques, another marker of neurodegeneration, by Congo red staining or immunoblot analysis using the anti-CT19 antibody recognizing the amyloid precursor protein (APP) carboxy-terminal C99 and C83 fragments [25] in the brains of HIV-1 Tg rats or WT. The number and size of amyloid plaques, stained with Congo red, were significantly elevated in the cerebral cortex of HIV-1 Tg rats compared to those of WT (Fig 4A and Figures E-G in S1 File), despite a relatively small number of amyloid plaques, compared to the amyloid plaques frequently observed in autopsied brain specimens from Alzheimer disease patients. In addition, the immuno-reactive levels of amyloid C-terminal fragment C99, which is critical for producing toxic amyloid-β peptides (e.g., Aβ1–42) [25, 26], were significantly elevated (i.e., by more than 5-fold) in the brains of HIV-1 Tg rats compared to WT (Fig 4B). However, under our conditions, the relatively less-toxic C83 amyloid fragment [26] was not clearly observed in the HIV-1 Tg and WT rats. Consistently, IHC staining (Fig 4C) and immunoblot analysis (Fig 4D) with the anti-β-amyloid antibody showed significantly increased levels of β-amyloid in the brains of HIV-1 Tg rats compared to the WT control.

### Neurodegeneration through hyperphosphorylated tau in HIV-1 Tg rats

**Fig 5:**
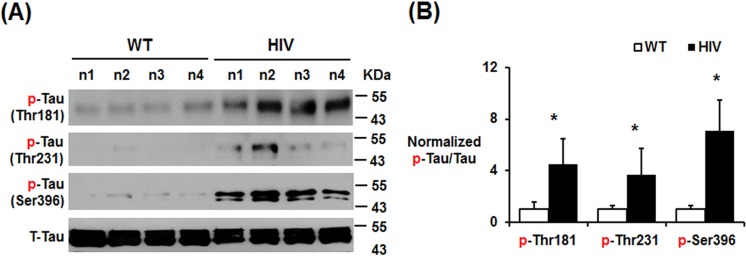
Increased tau hyperphosphorylation in the hippocampus of HIV-1 Tg rats. (A) Immunoblots for detecting tau phosphorylation at indicated amino acids to show different levels of tau hyperphosphorylation in WT and HIV-1 Tg rats. (B) Densitometric results for the immunoblots (n = 4/group) are presented. *P < 0.05.

Elevated β-amyloid can stimulate tauopathy and hyperphosphorylation of tau has been associated with changes in microtubule assembly to produce neurofibrillary tangles (NFTs) and neurodegeneration in HIV-1 infection [20, 27] as well as many other neurological diseases including Alzheimer’s and Parkinson’s disease [28–32]. In case of HIV infection, phosphorylation of tau (p-tau) is generally found in the hippocampus and entorhinal cortex before p-tau spreads to surrounding areas [33], which mirrors the pattern observed in Alzheimer disease individuals. Thus, we determined the levels of hyperphosphorylated tau in HIV-1 Tg and WT rats by immunoblot analysis. The levels of phosphorylated-tau (e.g., p-Thr181, p-Thr231, and p-Ser396) were markedly elevated in the hippocampus of HIV-1 Tg rats compared to those of the WT (Fig 5). However, hippocampal total tau levels were not changed in HIV-1 Tg rats (Fig 5), similar to the elevated p-tau proteins with little change in the total tau in HIV-1 infected patients and gp120 Tg mice [34]. Therefore, significantly elevated hyperphosphorylated tau (Fig 5B) is likely to produce NFTs and cause death of neuronal cells [27], resulting in neurodegeneration in HIV-1 Tg rats.

### Activation of CDK5 and apoptosis-related MAPKs in HIV-1 Tg rats

**Fig 6:**
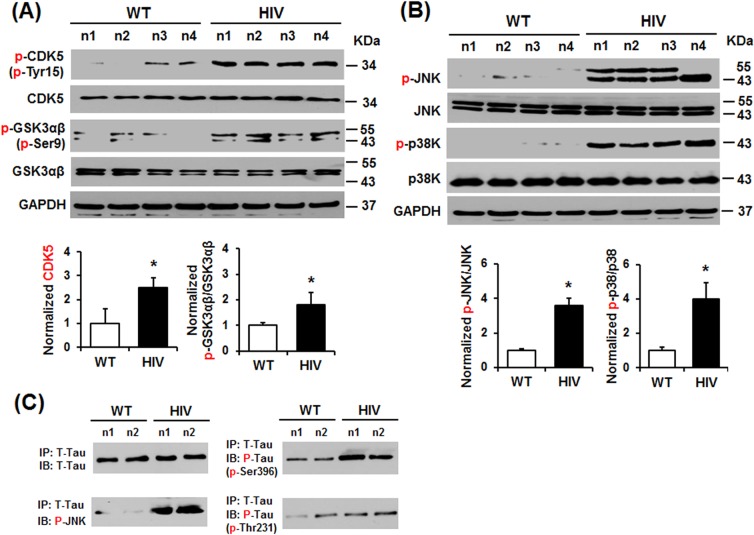
Activation of different protein kinases and JNK-mediated tau phosphorylation in the hippocampus of HIV-transgenic rats. (A, B) Representative immunoblots of hippocampal lysates from WT and HIV-1 Tg rats with the specific antibody to p-CDK5-Tyr15, CDK5, p-GSK3αβ-Ser9, GSK3αβ, p-JNK, JNK, p-p38K, p38K, or GAPDH, as indicated, are presented. Densitometric results for the immunoblots (n = 4/group) are presented. *P < 0.05. (C) Representative immunoblots of immunoprecipitated tau from WT and HIV-1 Tg rats with the specific antibody to p-JNK, p-tau-Ser396, p-tau-Thr231, or total tau (t-tau) are shown to demonstrate p-JNK binding to tau and hyperphosphorylation of indicated amino acids of tau in HIV-1 Tg rats.

Because of the increased amounts of hyperphosphorylated tau, we further investigated the role and levels of the major tau-phosphorylating protein kinases such as CDK5, GSK-3αβ, and MAPKs [35] including the apoptosis-related JNK and p38K. Immunoblot results revealed that the amounts of activated p-CDK5-Tyr15 (Fig 6A, top panel) and inactive p-GSK-3αβ-Ser9 (third panel) were significantly increased in HIV-1 Tg rats compared to those of WT. In addition, the levels of activated p-JNK (Fig 6B, top panel) and p-p38K (third panel) were significantly elevated (i.e., by more than 3- or 4-fold) in HIV-1 Tg rats. These results indicate that activated p-CDK5, p-JNK and p-p38K are important in tau phosphorylation in HIV-1 Tg rats. Since the role of JNK in tau phosphorylation is not clearly established, compared to CDK5 and GSK-3αβ, we performed additional experiments to further demonstrate its role in tau phosphorylation in HIV-1 Tg and WT rats. Immunoprecipitation of tau protein followed by immunoblot analysis revealed similar levels of immunoprecipitated total tau (Fig 6C, upper left panel). Our results showed that activated p-JNK appears to bind to immunoprecipitated tau (lower left panel) and thus phosphorylates at Ser396 and Thr231, respectively, as designated (right panels). These results indicate that elevated hyperphosphorylated p-tau in HIV-1 Tg rats can promote formation of NFTs and neuronal cell death, ultimately leading to neurodegeneration in the HIV-1 Tg rats, as reported previously [36, 37].

### Elevated production of inflammatory cytokines in HIV-1 Tg rats

**Fig 7:**
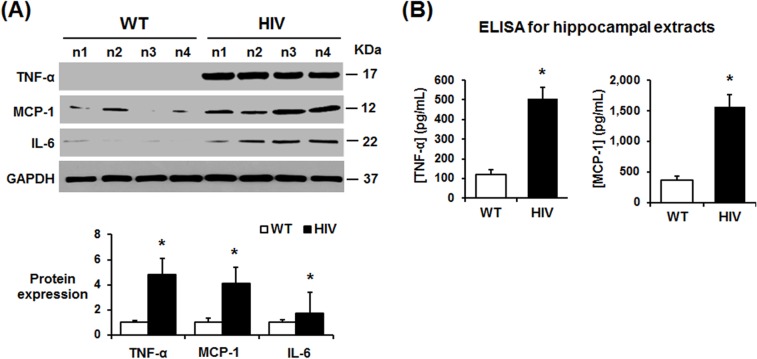
Increased proinflammatory cytokines in the hippocampus of HIV-1 Tg rats. (A) Representative immunoblots of hippocampal lysates from WT and HIV-1 Tg rats with the specific antibody to TNF-α, MCP-1, IL6, or GAPDH, as indicated. Densitometric quantitation of each indicated protein relative to GAPDH is shown (n = 4/group), *P < 0.05. (B) ELISA results for TNF-α and MCP-1 in the hippocampal lysates from WT and HIV-1 Tg rats are presented (n = 4/group), *P < 0.05.

Chronic inflammation with increased levels of proinflammatory cytokines and activated microglia are known to be potential mediators of neurodegeneration in HIV-1 infected people [38, 39]. The elevated pro-inflammatory cytokines such as TNF-α can also activate stress-activated MAPKs, including p-JNK [40], which can phosphorylate tau at multiple sites [35]. Therefore, the expressed amounts of TNF-α, MCP-1, and IL-6 in the hippocampus lysates from HIV-1 Tg and WT rats were evaluated by immunoblot analysis or ELISA. Immunoblot analyses (Fig 7A) and ELISA (Fig 7B) showed significantly elevated levels of all three cytokines in the hippocampal lysates from the HIV-1 Tg rats than those of the WT.

### Increased oxidative stress in HIV-1 Tg rats

**Fig 8:**
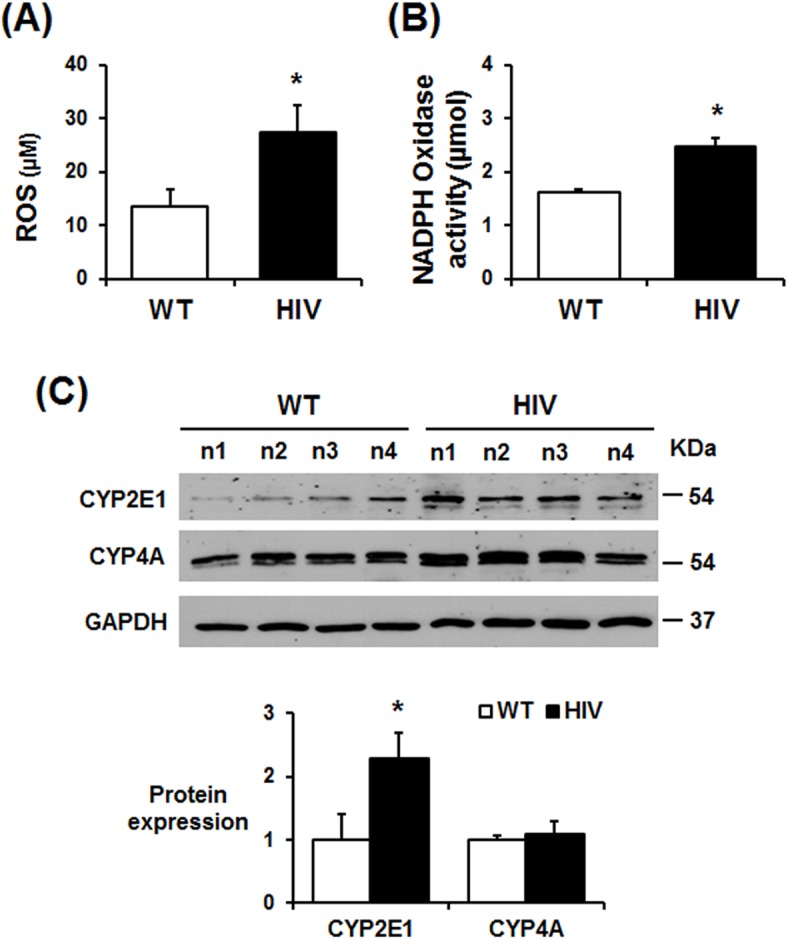
Increased oxidative stress, NADPH oxidase and CYP2E1 in the hippocampus of HIV-1 Tg rats. (A, B) Plasma ROS levels (A) and hippocampal NADPH oxidase activity in WT and HIV-1 Tg rats (n = 4/group), *P < 0.05. (C) Representative immunoblots of hippocampal lysates from WT and HIV-1 Tg rats with the specific antibody to CYP2E1, CYP4A, or GAPDH, as indicated. Densitometric quantitation of each indicated protein relative to GAPDH is also shown (n = 4/group), *P < 0.05.

Viral infections including HIV-1 usually elevate oxidative stress, which is known to promote HIV-related disease progression [41–43]. Therefore, we determined the relative levels of plasma ROS in HIV-1 Tg rats and WT. As expectedly, the amounts of ROS, measured by DCFH-DA fluorescence method, were significantly elevated in HIV-1 Tg rats (Fig 8A). In addition, the NADPH oxidase activities in the hippocampal extracts were significantly increased in HIV-1 Tg rats compared to WT rats (Fig 8B). The hippocampus levels of cytochrome P450-2E1 (CYP2E1), which is known to be involved in producing reactive oxygen species [44] and nitroxidative protein modifications [45, 46], were increased in HIV-1 Tg rats while other P450 isozymes, such as CYP4A, which is also known to produce ROS [47, 48], did not seem to be increased in HIV-1 Tg rats compared to those of WT counterparts (Fig 8C).

**Fig 9:**
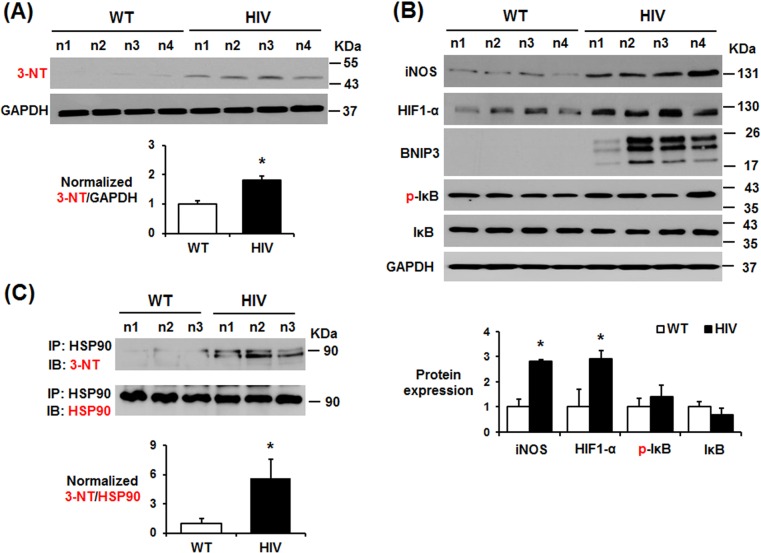
Increased nitroxidative stress marker proteins in the hippocampus of HIV-1 Tg rats. (A, B) Representative immunoblots of hippocampal lysates from WT and HIV-1 Tg rats with the specific antibody to 3-NT, iNOS, HIF1-α, BNIP3, p-IκB, IκB, or GAPDH, as indicated. Densitometric quantitation of each indicated protein relative to GAPDH is shown (n = 4/group). *P < 0.05. (C) Representative immunoblots of immunoprecipitated Hsp90 from WT and HIV-1 Tg rats with the specific antibody to 3-NT or HSP90 are shown to demonstrate nitration of Hsp90 in HIV-1 Tg rats.

Consistently, the amounts of nitrated proteins, determined by anti-3-nitroTyr (3-NT) antibody (Fig 9A), inducible nitric oxide synthase (iNOS), hypoxia-inducible factor-1α (HIF-1α), an upstream transcription factor of iNOS [49–51], and BNIP3, a downstream target of HIF-1α [51], were significantly elevated in the HIV-1 Tg rats compared with WT (Fig 9B). However, the levels of NF-κB-associated proteins including Iκ-B and its phospho-Iκ-B (p-IκB) did not significantly differ between the HIV-1 Tg and WT rats (Fig 9B). Peroxynitrite and protein nitration appear to be important for HIV-mediated neurodegeneration, since the rates and severity of HIV-mediated dementia correlated with the levels of gp41 and iNOS in HIV-1 infected patients [52, 53]. To further support the increased levels of nitrated proteins in HIV-1 Tg rats (Fig 9A), nitration of Hsp90 in HIV-1 Tg rats was evaluated as an example of nitrated proteins by employing immunoprecipitation of total Hsp90 followed by immunoblot for 3-NT or Hsp90, since nitrated Hsp90 can directly induce cell death [54]. The immunoprecipitation and immunoblot experiments revealed increased nitration of Hsp90 in HIV-1 Tg rats compared to the WT rats (Fig 9C). These results suggest that nitroxidative stress with increased nitrated proteins, including Hsp90, along with elevated p-Tau in HIV-1 Tg rats are likely to promote apoptosis of neuronal cells, contributing to NFT production and neurodegeneration.

**Fig 10:**
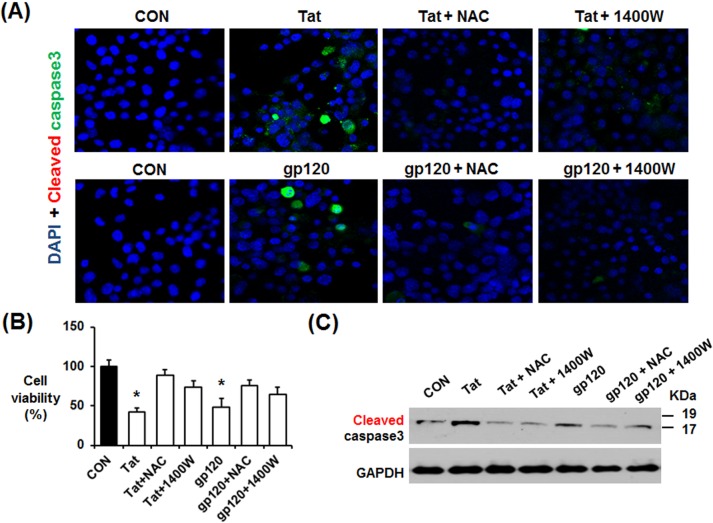
Causal role of increased nitroxidative stress in neuronal cell death exposed to recombinant Tat or gp120 protein. (A-C) Representative images of confocal microscopy (A) of cleaved caspase-3, MTT assay (B) and immunoblot assay with anti-caspase-3 (C) to show the apoptosis rates of neuro-2A cells exposed to the recombinant HIV protein Tat or gp120 in the absence or presence of NAC or 1400W. (B) MTT cell viability analysis to determine the cell death rates of neuro-2A cells exposed to the recombinant Tat or gp120 in the absence or presence of 1 mM NAC or 20 μM 1400W. GADPH was used as a loading control.

These results suggest that HIV-1 proteins elevate nitroxidative stress, leading to neuronal cell death and neurodegeneration. To directly study the causal role of increased nitroxidative stress in neuronal cell death, we studied the effects of an antioxidant NAC and iNOS inhibitor 1400W on the rates of apoptosis of neuro2A cells exposed to the recombinant HIV-1 Tat or gp120 protein, which is known to increase oxidative stress and cell death [55]. Confocal microscopy revealed that both HIV-1 Tat and gp120 increased cell death, as evidenced by elevated levels of cleaved (activated) caspase-3 (Fig 10A). Both NAC and 1400W treatments significantly suppressed Tat- or gp120-mediated cell death. Consistently, MTT cell viability assays also showed that both HIV proteins increased cell death, which was significantly prevented by co-treatment with NAC or 1400W (Fig 10B). Similar results of cleaved caspase-3 were observed by immunoblot analysis (Fig 10C). These results indicate that HIV-mediated increased nitroxidative stress plays a critical role in causing neuronal cell death, contributing to neurodegeneration.

## Discussion

Epidemiological and clinical studies revealed that people infected with HIV-1 are more susceptible to various disease states than the non-infected people. For instance, the HIV-1 infected people shows signs of severe immune dysfunction or dysregulation [2] with abnormalities in the CNS with neurocognitive decline and many peripheral tissues such as interstitial pneumonia [3], tubulointerstitial nephritis [4], intestinal disturbance [5], heart disease [6], and liver diseases often resulting in death [7]. The usage of highly active anti-retroviral therapy (HAART) significantly improved the overall death rate and severity of HIV-1 related disorders, although it can increase the prevalence of HAND [8, 56, 57].

The HAND represents HIV-associated neurocognitive disorders with various severity such as AIDS dementia complex (ADC) also known as HIV dementia and HIV-associated dementia (HAD), mild neurocognitive disorder (MND), etc. However, to our knowledge, the underlying mechanisms for promoting HAND and/or HAD have not been completely understood. Therefore, a few in vitro and in vivo experimental gp120- or Tat-Tg mouse [58, 59] or HIV-1 Tg rat [60] models have been developed to simulate HIV-1 infected human conditions and to study the mechanisms of neurological deficits. In fact, the 10-month old Tg mice with over-expressed HIV/gp120 showed signs of cognitive abnormalities with increased neuronal cell death, tau-phosphorylation, and increased gliosis compared to the age-matched WT mice [58]. These animal results are in agreement with the increased neurological disorder with markedly elevated levels of phospho-tau and abnormal NFTs in HIV-infected people with HAND compared to healthy normal individuals or HIV-1 infected people without HAND [58], similar to those of other reports [20, 33, 34, 61], although other reports suggested that phospho-tau-Thr181 does not appear to be important in neurodegeneration in HIV-infected people [62, 63]. These conflicting results indicate that the role of tau and/or phospho-tau in causing HAND and HAD is still poorly established and thus needs further studies. However, normal or unchanged levels of tau [64] or p-tau-Thr181 level [62, 63] should not necessarily rule out the important role of tau and/or phospho-tau, since many other amino acids of tau [33], such as p-tau-Ser202 [33, 34], p-tau-Thr231 (Fig 5 of this study) and p-tau-Ser396/Ser404 [34] could be phosphorylated by various protein kinases, including CDK5, GSK-3β, and MAPKs, that are activated in HIV-infected patients [34], gp120 Tg mice [34], and HIV-1 Tg rats (Figs 5 and 6). In fact, the elevated levels of p-tau with little changes in total tau amounts in HIV-infected patients and gp120 Tg mice [34] along with prevention of neurobehavioral deficits by the selective CDK5 inhibitor roscovitine [34] and our current results (Fig 5) strongly suggest that phospho-tau status may be more important than total tau levels in promoting NFT and neurodegeneration. Further, our results (Fig 6) also showed that activated p-JNK, used as an example, actually interacts and phosphorylates tau at Thr231 and Ser396. Based on these facts and reasons, it is highly desirable to study the status of many other phosphorylation sites of tau, instead of only determining the levels of total tau and/or p-tau-Thr181 in making final conclusion, since tauopathy seems more closely correlated with the dementia status and a better predictor of cognitive function than amyloid deposits [65].

In HIV/Tat over-expressed mice [59], increased neuronal death and elevated infiltration of activated monocytes and T lymphocytes were observed. These results indicate an important role of HIV proteins in causing neurotoxicity and that the levels of phospho-tau are likely to indirectly reflect the severity of neuropathology with cognitive decline, as reported [66]. In addition, the HIV-1 Tg rats, produced by using the NL4-3 gag/pol HIV-1 transgene and contain all viral genes except for *gag* and *pol* genes [60], have distinct cataracts at birth but otherwise appear healthy until certain ages. However, HIV-1 Tg rats developed many clinical manifestations of AIDS by 5–9 months of age including weight loss, neuroglial abnormalities, and respiratory difficulties that progresses over time [60]. Consistently, significantly smaller body weights were observed in HIV-1 Tg rats than the aged- and gender-matched WT (Figure A in S1 File), possibly suggesting reduced food intake and/or abnormal energy production and utilization. In addition, HIV-1 Tg rats show signs of significant behavioral and cognitive deficits when they become 5-month old [36, 37], while they are known to have increased nitroxidative stress and more susceptible to barrier dysfunction and neuronal damage [41–43, 67]. Despite many reports, the underlying molecular mechanisms for various pathological conditions are poorly understood except for general descriptions about increased inflammation, oxidative stress and the cognitive and behavioral deficits observed in 5-month old HIV-1 Tg rats compared to the corresponding control WT [36, 37]. For instance, to our knowledge, the source of increased nitroxidative stress, change in the cell signaling pathways, the role of MAPK activation in tau binding and phosphorylation have not been evaluated in HIV-1 Tg rats. Therefore, this study was aimed to specifically investigate the source and levels of nitroxidative stress and its role in activating MAPKs for tau hyperphosphorylation, contributing to neuronal cell death and neurodegeneration in 5-month old HIV-Tg rats compared to those of WT. We particularly focused on the cell death-associated JNK to evaluate its binding to tau and its role in tau hyperphosphorylation, nitration of Hsp90 and upregulation of Bax in HIV-Tg versus WT rats.

Increased oxidative stress can stimulate the pathogenesis of HIV-1-related disorders [68–70]. Agrawal and colleagues also reported that HIV-1 related neurotoxicity can be prevented by gene delivery of antioxidant enzymes such as glutathione peroxidase and superoxide dismutase into the brain [71]. Additionally, suppression of antioxidant NRF2 using specific siRNA aggravated the HIV/gp120-mediated oxidative stress and neurocognitive decline [72]. These results indicate a critical role of increased oxidative stress in HIV-1-mediated neurotoxicities. Consistently, our results (Fig 10) also showed that HIV protein Tat or gp120 increased neuronal cell death, which can be prevented by an antioxidant or a specific iNOS inhibitor, suggesting an important role of nitroxidative stress and protein nitration in causing neuronal cell death. However, the source of increased oxidative stress and its role in promoting tauopathy and neurodegeneration in HIV-1 Tg rats have not been fully characterized. Buch and his colleagues reported that HIV/gp120 activates NADPH-oxidase in the neuronal tissues [73, 74]. In addition, HIV/gp120 elevated CYP2E1 levels and caused apoptosis of cultured astrocytes through oxidative stress-related events [75]. The CYP2E1-related oxidative stress and apoptosis of astrocytes were significantly blocked by the CYP2E1 inhibitor diallyl sulfide and specific siRNA, suggesting an important role of CYP2E1 in promoting oxidative stress and apoptosis [75]. Our results (Fig 8) revealed that both NADPH-oxidase activity and CYP2E1 level were elevated in the hippocampus of the HIV-1 Tg rats than those of WT. Therefore, elevated NADPH-oxidase and CYP2E1, known to promote oxidative stress [44–48], were likely to increase the measured amounts of ROS (Fig 8), which can produce a potently-toxic agent peroxynitrite in the presence of NO, possibly produced by elevated iNOS (Fig 9B). Elevated peroxynitrite can nitrate many proteins, leading to mitochondrial dysfunction and cell death [45]. In fact, elevated levels of nitrated proteins, determined by immunoblot using the anti-3-NT antibody, were observed in HIV-1 Tg rats compared to the WT (Fig 9A). Immunoprecipitation followed by immunoblot analysis further confirmed nitration of Hsp90, evaluated as an example of nitrated proteins in HIV-1 Tg rats (Fig 9C), contributing to neuronal cell death, as originally reported for increased apoptosis of cultured cells [54]. Based on the results (Fig 9), it is likely that many other proteins could be also nitrated and thus contribute to mitochondrial dysfunction and organ damage, as recently reviewed [45]. Furthermore, elevated HIF1-α and BNIP3, involved in executing apoptosis [51], could have stimulated neuronal cell death (Figs 1–3) in HIV-1 Tg rats.

Increased presence of neurofibrillary tangles (NFT) composed of hyperphosphorylated tau represents another hallmark of HAND observed in people infected with HIV [27]. Elevated tau hyperphosphorylation in HIV-infected people or experimental animals likely results from various protein kinases activated by HIV-1 viral proteins [58] or pro-inflammatory cytokines [40] that are known to increase oxidative stress. In the context of HIV-1 infection, hyperphosphorylated tau is generally found in the hippocampus and entorhinal cortex before it later spreads to surrounding areas. GSK-3β and CDK5 are have been implicated in the phosphorylation of tau at various amino acid residues [76, 77]. In our study, hyperphosphorylation of tau was increased in the hippocampus of HIV-1 Tg rats compared with the WT, as similar to the previous reports [76, 77]. In addition, we observed elevated phosphorylation (inactivation) of Ser9 of GSK-3β with phosphorylation (activation) of Tyr15 of CDK5 in the hippocampus of HIV-1 Tg rats (Fig 6A). These results indicate that GSK-3β may not be as important as CDK5 in tau phosphorylation in HIV-1 Tg rats. Increased oxidative stress and inflammatory cytokines such as such as TNF-α are also known to activate (phosphorylate) MAPKs such as cell-death associated JNK and p38K [23, 40] possibly through suppression of their respective phosphatases [78]. JNKs and p38K are the major brain MAPKs and critically important in neuronal cell death and neurodegeneration through phosphorylation of tau [35, 79, 80] and amyloid precursor protein followed by the production of a membrane-associated amyloid C99 fragment [25]. In theory, activated p-JNK or p-P38K can phosphorylate tau at different amino acids such as Thr181, Ser199, Ser202, Thr205, Thr231, Ser396, Ser404, etc [35]. The levels of activated p-JNK and p-p38K were significantly increased in the hippocampus of HIV-1 Tg rats compared to WT rats, as similar to the recent results [81]. Immunoprecipitation followed by immunoblot analysis results further confirmed JNK interaction with tau and its phosphorylation at Thr231 and Ser396 (Fig 6C), although other amino acids can be also phosphorylated by activated p-JNK or p-p38K (Fig 6B). The elevated hyperphosphorylated tau is likely to promote neuronal cell death via tauopathy with tau-associated NFTs [20, 27] along with production of APP-derived toxic C-terminal C99 fragment (Fig 4), ultimately leading to neurodegeneration in HIV-1 Tg rats. In addition, immunoblot analyses showed elevated levels of cleaved caspase-3 and pro-apoptotic Bax protein in the hippocampus of HIV-1 Tg rats (Fig 2) compared to those of healthy WT. These results are consistent with death of neuron cells, as determined by cleaved caspase-3 and histological staining, in the hippocampus region of HIV-1 Tg rats. Similar to Alzheimer disease [31, 32], the JNK and/or p38K-dependent apoptosis pathway [23] along with increased phosphorylation of Tau (Fig 6C in this study) and p-Thr668 of APP [26], all of which can stimulate neuronal cell death in the hippocampus region, contributing to cognitive and behavioral deficits in HIV-1 Tg rats [36, 37, 81]. Although we have not evaluated the role of activated p-p38K in tau-phosphorylation in our study, it is likely that its role could be as similar as that of p-JNK. Therefore, suppression of activated p-JNK (and/or p-p38K) can be an excellent target for preventing or treating various neurodegenerative diseases, as recently reviewed [40, 82].

Immune dysfunction and abnormal inflammatory processes represent a hallmark of HIV-1 infection, which may further increase the sustained imbalance of cellular redox state, as observed in HIV-1 infected patients. MCP-1 appears to be one of the most important chemokines in the pathogenesis of HIV infection, and its levels are elevated in the brain and CSF of patients with HIV-associated dementia [83]. Previous studies showed that inflammatory cytokines such as IFN-γ, TNF-α, and IL1β were elevated in the HIV-1 Tg rats compared to WT rats [81], suggesting chronic inflammation in the HIV-1 Tg rats. In agreement with these reports, our immunoblot analysis and ELISA (Fig 7) also showed that the levels of TNF-α, IL6, and MCP-1 were significantly higher in the hippocampus of HIV-1 Tg rats compared to those of the WT. Thus, it is likely that increased MCP-1 stimulates infiltration of activated monocytes and T lymphocytes into the brain in HIV-1 Tg rats, as similar to the mice with over-expressed HIV/Tat proteins [71]. Nonetheless, elevated cellular nitroxidative stress and inflammatory responses with activated JNK/p38K and protein nitration may be the major triggering mechanisms for increased neurodegeneration in HIV-1 Tg rats.

**Fig 11:**
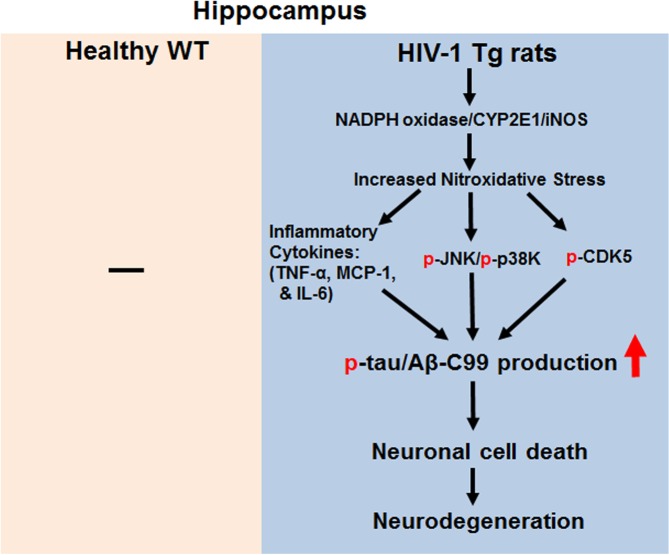
Schematic mechanisms for increased neurodegeneration in HIV-1 Tg rats.

In conclusion, our mechanistic studies have shown new results that elevated CYP2E1, NADPH oxidase, and iNOS, all of which can produce reactive oxygen and nitrogen species, respectively, play a role in producing increased production of peroxynitrite, which can nitrate Tyr residues of many proteins [45, 78], including Hsp90 (Fig 9), which can stimulate cell death upon nitration [54]. In parallel, the levels of pro-inflammatory cytokines/chemokines were elevated in the hippocampus of HIV-1 Tg rats. All these pro-oxidative and pro-inflammatory changes can stimulate CDK5 and JNK/p38K with increased levels of tau hyperphosphorylation with abnormal NFTs, toxic amyloid C99 fragment, pro-apoptotic Bax and cleaved (activated) caspase-3, contributing to neuronal cell death and neurodegeneration in HIV-1 Tg rats, as summarized (Fig 11). Based on the elevated nitroxidative stress and chronic inflammation, it is likely that similar molecular mechanisms can take place for increased neuronal cell death and neurodegeneration often observed in HIV-infected people.

## Supporting Information

S1 File Comparison of the body weight and neuronal cell death in five-month-old WT and HIV-1 Tg rats. Relative body weight of WT and HIV-1 Tg rats. (n = 8/group), *P < 0.05 **(Figure A).** Representative IHC staining for GFAP in the cerebral cortex, hippocampal CA1 region and dentate gyrus of the WT and HIV-1 Tg rats **(Figure B).** Summary of the number of NeuN-positive or GFAP-positive cells in the cerebral cortex, hippocampal CA1 region and dentate gyrus of the WT and HIV-1 Tg rats. *P < 0.05 **(Figure C).** Increased apoptosis of neuronal cells in HIV-1 Tg rats. (Left) Representative immunoblots of hippocampal lysates from WT and HIV-1 Tg rats with the respective antibody to NeuN, GFAP, or GAPDH, used as a loading control, as indicated. (Right) Similar immunoblot results were observed with another set (n = 4/group). Densitometric analysis summary of the immunoblots for NeuN or GFAP relative to GAPDH is shown below. *P < 0.05 **(Figure D).** Representative Congo red staining of β-amyloid in the cerebral cortex of the HIV-1 Tg rats with different magnifications, as indicated **(Figure E).** Representative IHC staining of β-amyloid in the cerebral cortex of a HIV-1 Tg rat with different magnifications, as indicated **(Figure F).** IHC staining of β-amyloid in the cerebral cortex of another HIV-1 Tg rat with different magnifications, as indicated **(Figure G). **
(PPTX)
