# Supplementary material for: Neuronal Cell Death and Degeneration through Increased Nitroxidative Stress and Tau Phosphorylation in HIV-1 Transgenic Rats
Source: PLoS One. 2017 Jan 20;12(1):e0169945. doi: 10.1371/journal.pone.0169945 (PMC5249108; doi:10.1371/journal.pone.0169945)
Supplement: S1 File — Relative body weight of WT and HIV-1 Tg rats. (n = 8/group), *P < 0.05 (Figure A). Representative IHC staining for GFAP in the cerebral cortex, hippocampal CA1 region and dentate gyrus of the WT and HIV-1 Tg rats (Figure B). Summary of the number of NeuN-positive or GFAP-positive cells in the cerebral cortex, hippocampal CA1 region and dentate gyrus of the WT and HIV-1 Tg rats. *P < 0.05 (Figure C). Increased apoptosis of neuronal cells in HIV-1 Tg rats. (Left) Representative immunoblots of hippocampal lysates from WT and HIV-1 Tg rats with the respective antibody to NeuN, GFAP, or GAPDH, used as a loading control, as indicated. (Right) Similar immunoblot results were observed with another set (n = 4/group). Densitometric analysis summary of the immunoblots for NeuN or GFAP relative to GAPDH is shown below. *P < 0.05 (Figure D). Representative Congo red staining of β-amyloid in the cerebral cortex of the HIV-1 Tg rats with different magnifications, as indicated (Figure E). Representative IHC staining of β-amyloid in the cerebral cortex of a HIV-1 Tg rat with different magnifications, as indicated (Figure F). IHC staining of β-amyloid in the cerebral cortex of another HIV-1 Tg rat with different magnifications, as indicated (Figure G). (PPTX) [file pone.0169945.s001.pptx]

## Slide 1
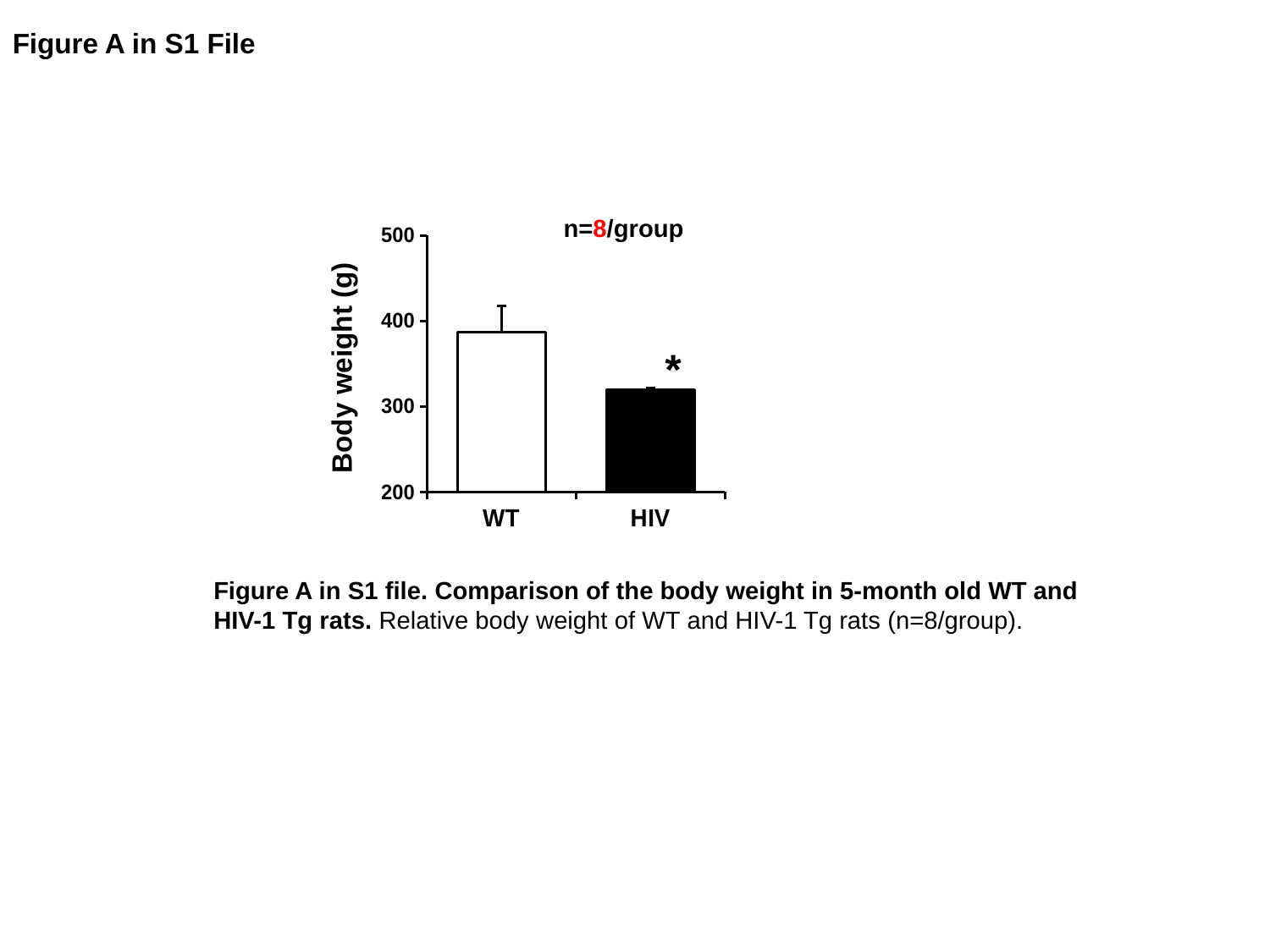

Figure A in S1 File
### Chart
| Category | |
|---|---|
| WT | 387.5 |
| HIV | 319.25 |n=8/group
*
Body weight (g)
Figure A in S1 file. Comparison of the body weight in 5-month old WT and HIV-1 Tg rats. Relative body weight of WT and HIV-1 Tg rats (n=8/group).

## Slide 2
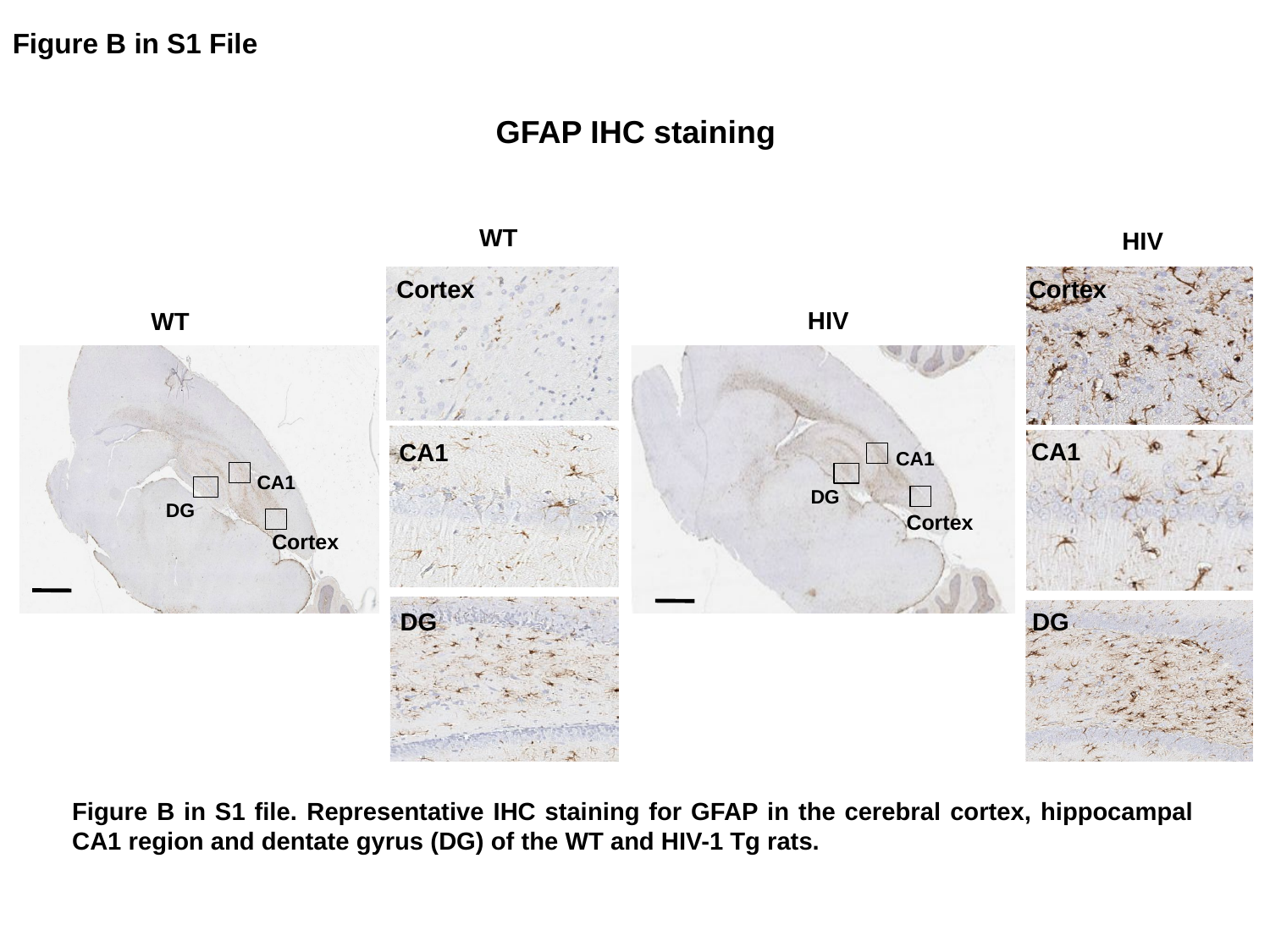

Figure B in S1 File
 GFAP IHC staining
 WT
 HIV
Cortex
Cortex
 HIV
 WT
CA1
CA1
CA1
CA1
DG
DG
Cortex
Cortex
DG
DG
Figure B in S1 file. Representative IHC staining for GFAP in the cerebral cortex, hippocampal CA1 region and dentate gyrus (DG) of the WT and HIV-1 Tg rats.

## Slide 3
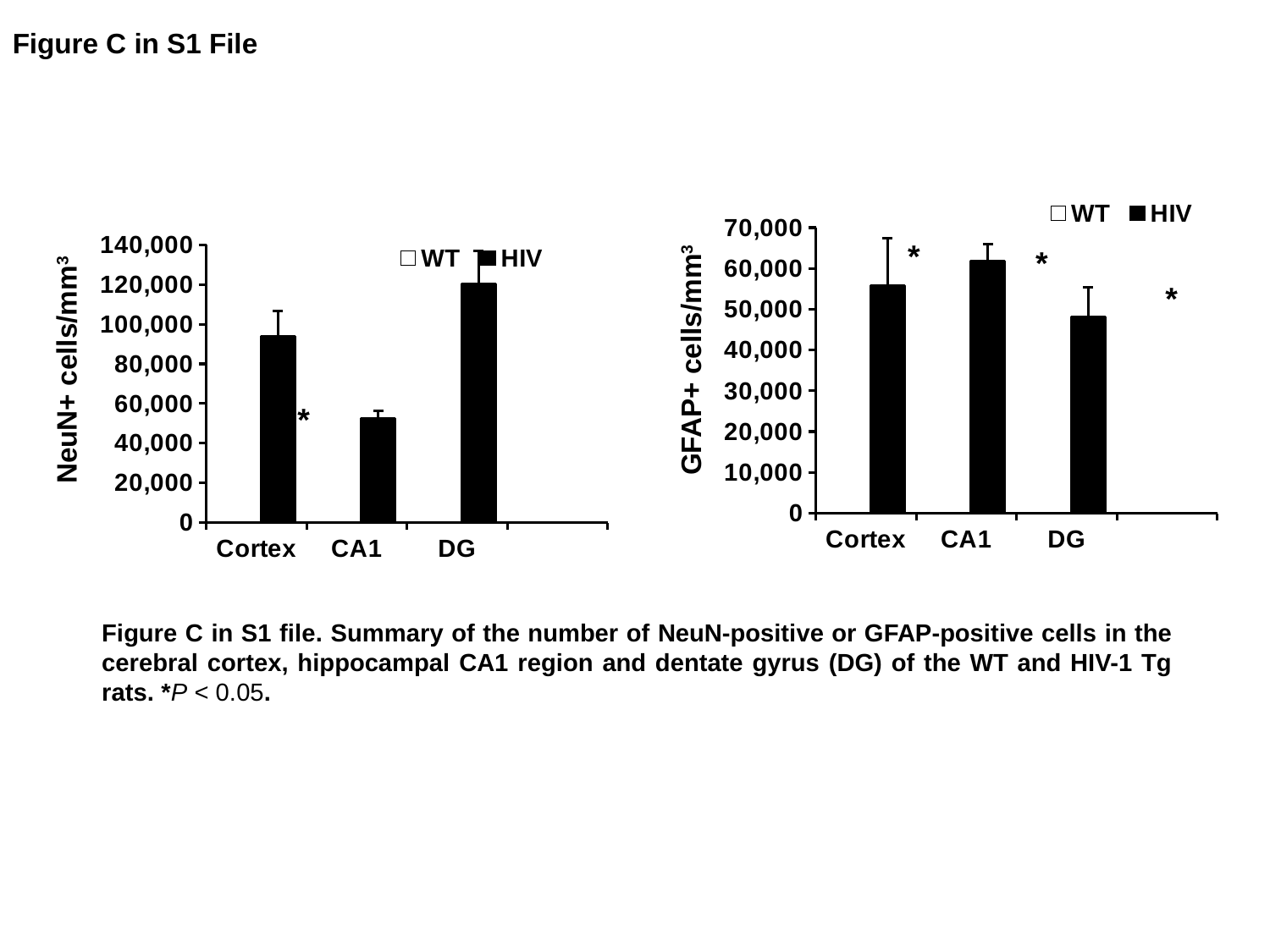

Figure C in S1 File
### Chart
| Category | WT | HIV |
|---|---|---|
| Cortex | 15416.666666666668 | 55856.48148148148 |
| CA1 | 29305.55555555556 | 61875.00000000001 |
| DG | 23958.333333333332 | 48171.296296296314 |
### Chart
| Category | WT | HIV |
|---|---|---|
| Cortex | 250555.5555555556 | 93888.88888888889 |
| CA1 | 73333.0 | 52314.0 |
| DG | 186666.6666666667 | 120416.66666666664 |*
*
*
GFAP+ cells/mm3
NeuN+ cells/mm3
*
Figure C in S1 file. Summary of the number of NeuN-positive or GFAP-positive cells in the cerebral cortex, hippocampal CA1 region and dentate gyrus (DG) of the WT and HIV-1 Tg rats. *P < 0.05.

## Slide 4
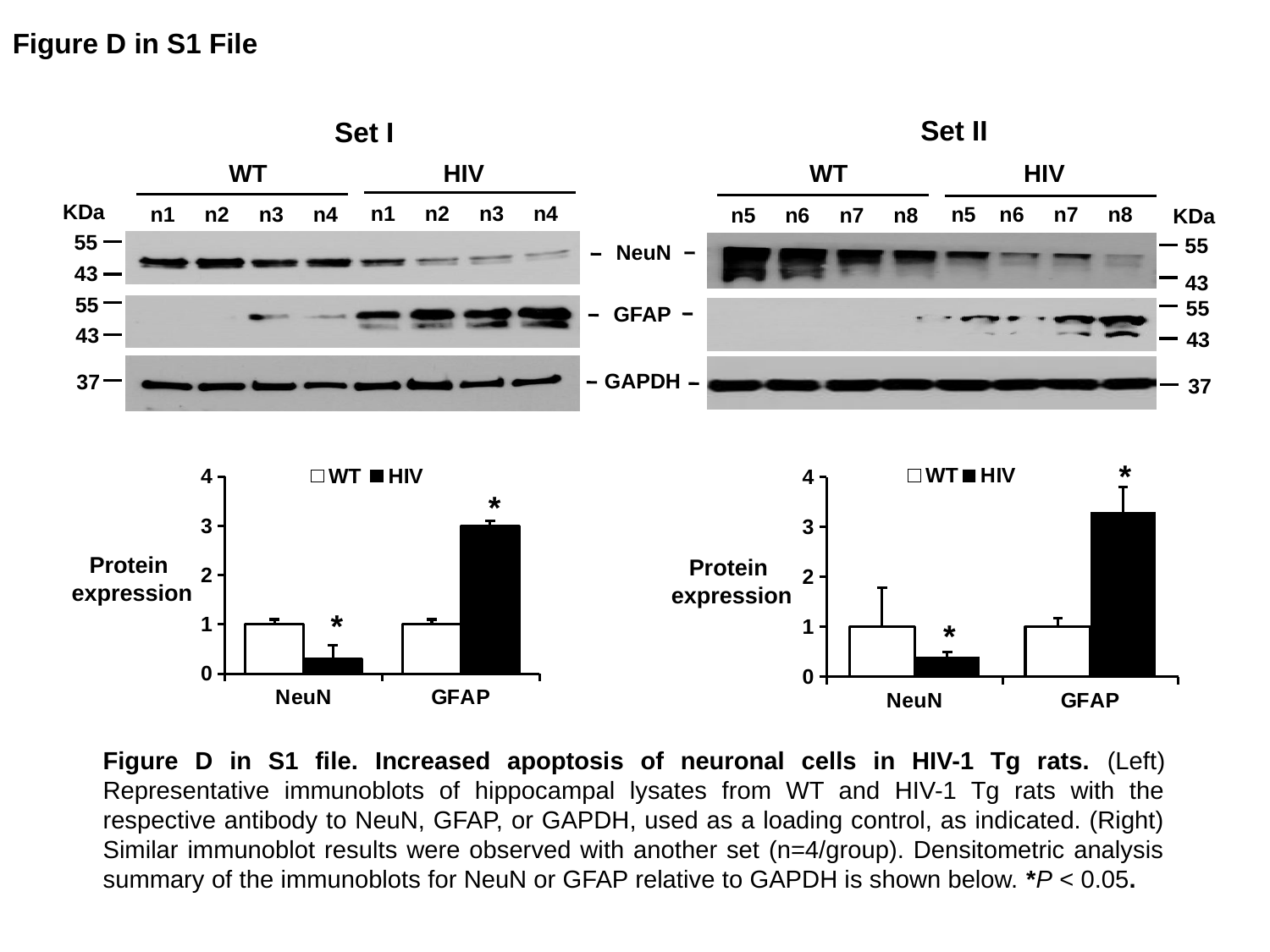

Figure D in S1 File
Set II
Set I
WT
HIV
WT
HIV
KDa
 n1 n2 n3 n4
 n5 n6 n7 n8
 n1 n2 n3 n4
 n5 n6 n7 n8
KDa
55
55
NeuN
43
43
55
55
GFAP
43
43
GAPDH
37
37
### Chart
| Category | WT | HIV |
|---|---|---|
| NeuN | 1.0 | 0.3 |
| GFAP | 1.0 | 3.0 |
### Chart
| Category | WT | HIV |
|---|---|---|
| NeuN | 1.0 | 0.4 |
| GFAP | 1.0 | 3.3 |*
*
Protein
expression
Protein
 expression
*
*
Figure D in S1 file. Increased apoptosis of neuronal cells in HIV-1 Tg rats. (Left) Representative immunoblots of hippocampal lysates from WT and HIV-1 Tg rats with the respective antibody to NeuN, GFAP, or GAPDH, used as a loading control, as indicated. (Right) Similar immunoblot results were observed with another set (n=4/group). Densitometric analysis summary of the immunoblots for NeuN or GFAP relative to GAPDH is shown below. *P < 0.05.

## Slide 5
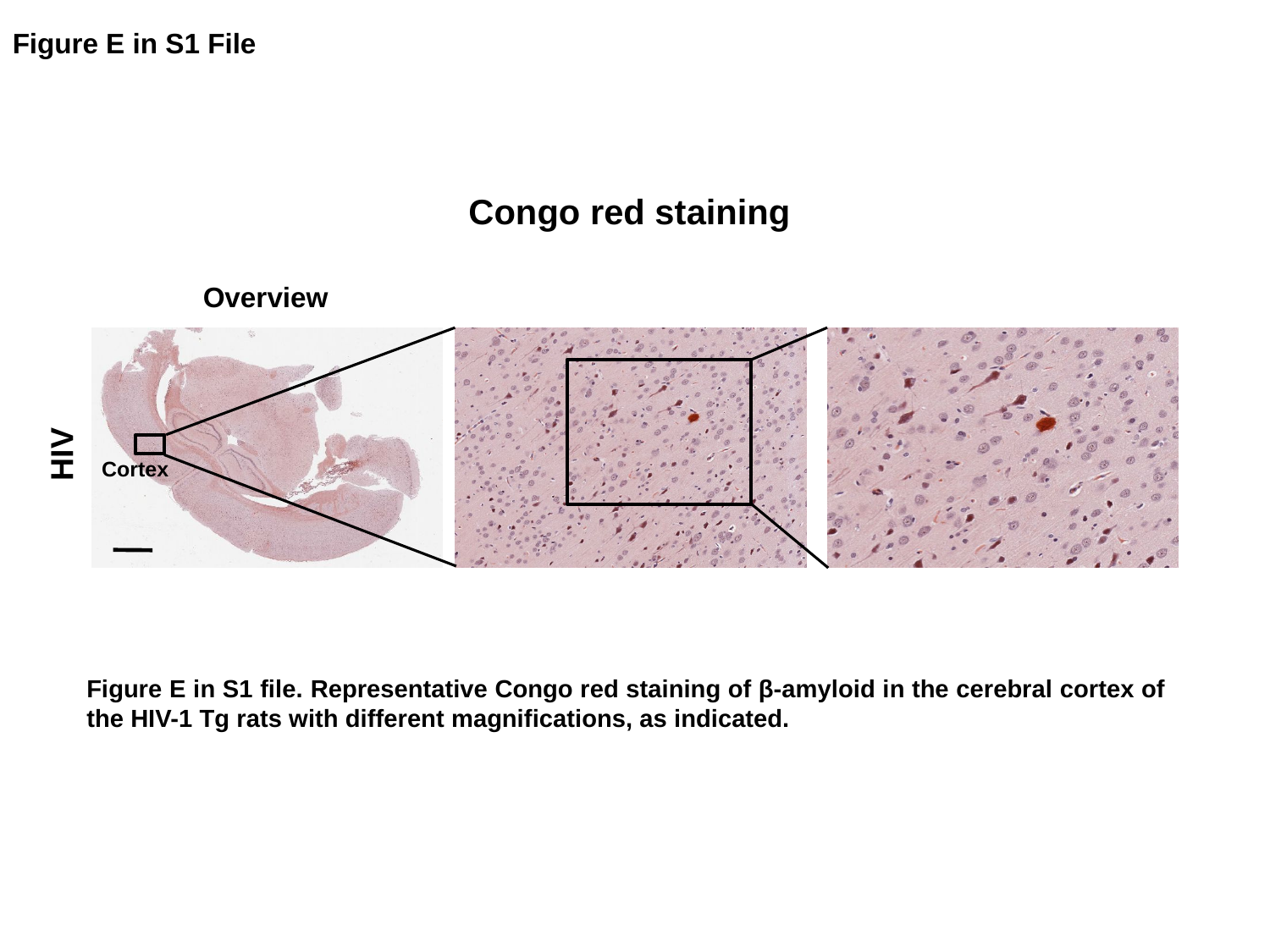

Figure E in S1 File
 Congo red staining
Overview
HIV
Cortex
Figure E in S1 file. Representative Congo red staining of β-amyloid in the cerebral cortex of the HIV-1 Tg rats with different magnifications, as indicated.

## Slide 6
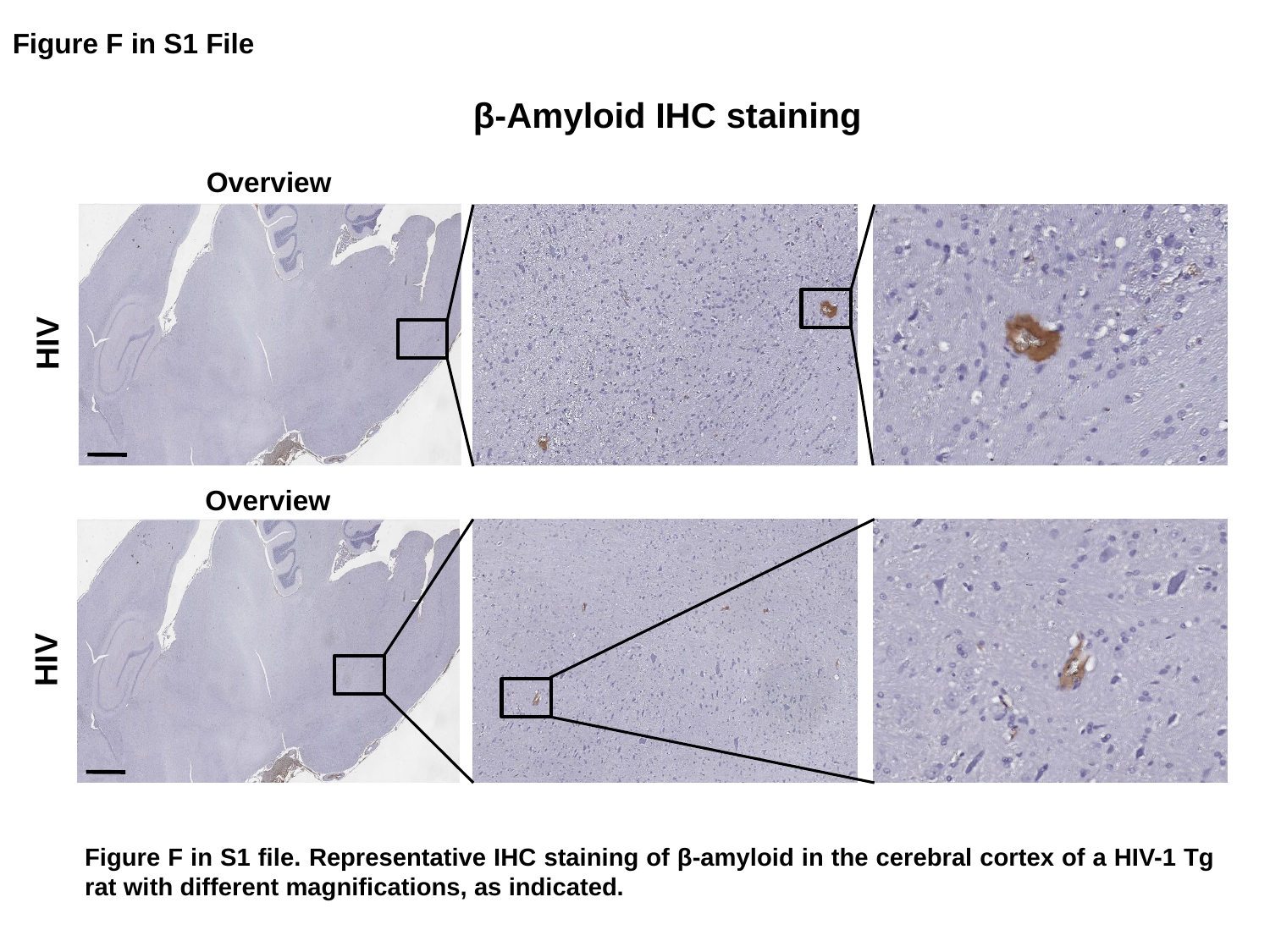

Figure F in S1 File
 β-Amyloid IHC staining
Overview
HIV
Overview
HIV
Figure F in S1 file. Representative IHC staining of β-amyloid in the cerebral cortex of a HIV-1 Tg rat with different magnifications, as indicated.

## Slide 7
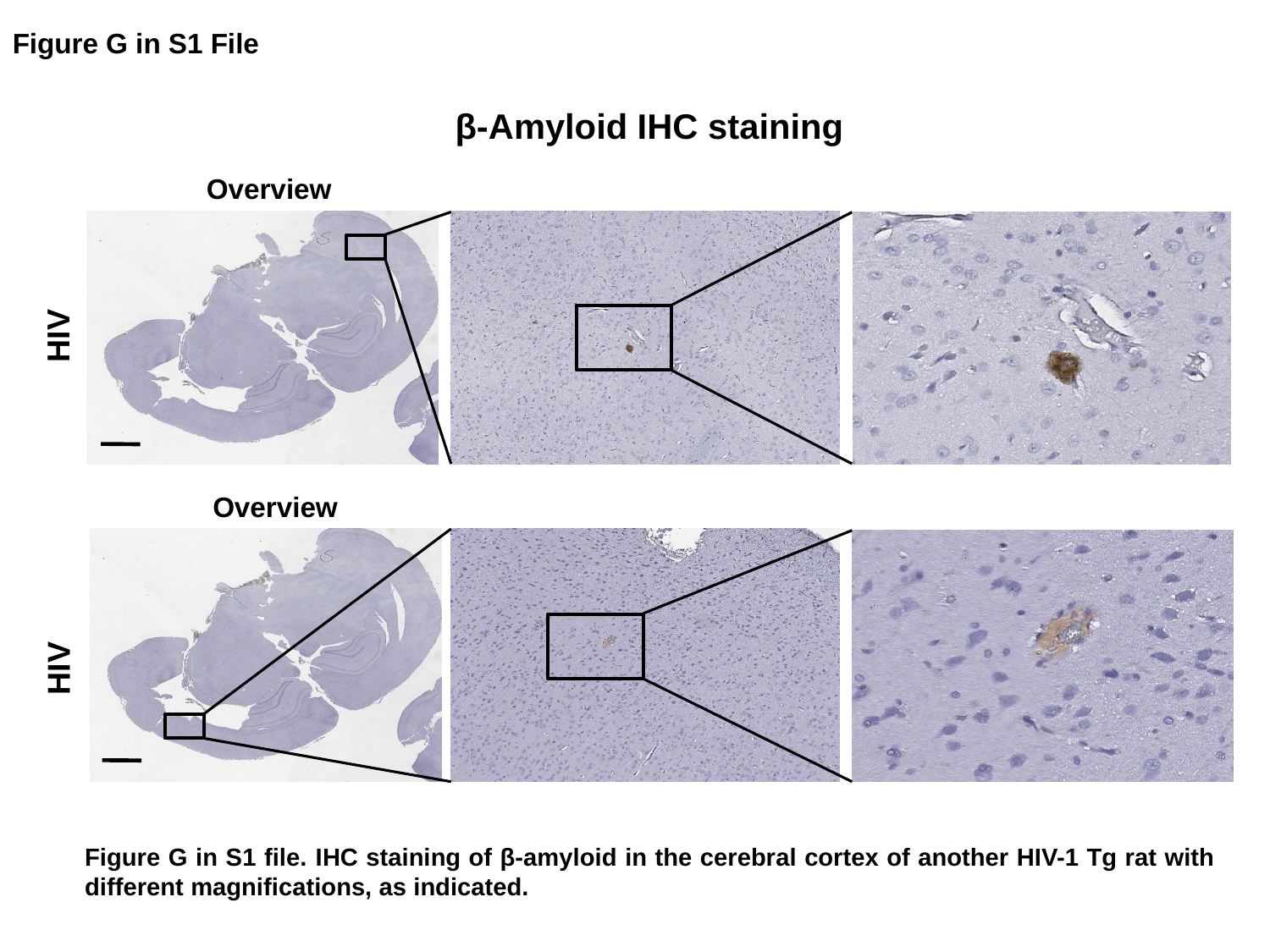

Figure G in S1 File
 β-Amyloid IHC staining
Overview
HIV
Overview
HIV
Figure G in S1 file. IHC staining of β-amyloid in the cerebral cortex of another HIV-1 Tg rat with different magnifications, as indicated.
